# ADAR2 induces reproducible changes in sequence and abundance of mature microRNAs in the mouse brain

**DOI:** 10.1093/nar/gku844

**Published:** 2014-09-26

**Authors:** Cornelia Vesely, Stefanie Tauber, Fritz J. Sedlazeck, Mansoureh Tajaddod, Arndt von Haeseler, Michael F. Jantsch

**Affiliations:** 1 Department of Chromosome Biology, Max F. Perutz Laboratories, University of Vienna, Dr. Bohr Gasse 9, A-1030 Vienna, Austria; 2 Center for Integrative Bioinformatics Vienna, Max F. Perutz Laboratories, University of Vienna, Medical University of Vienna, and University of Veterinary Medicine, Dr. Bohr Gasse 9, A-1030 Vienna, Austria; 3 Bioinformatics and Computational Biology, Faculty of Computer Science, University of Vienna, Währinger Straße 29, A-1090 Vienna, Austria

## Abstract

Adenosine deaminases that act on RNA (ADARs) deaminate adenosines to inosines in double-stranded RNAs including miRNA precursors. A to I editing is widespread and required for normal life. By comparing deep sequencing data of brain miRNAs from wild-type and ADAR2 deficient mouse strains, we detect editing sites and altered miRNA processing at high sensitivity. We detect 48 novel editing events in miRNAs. Some editing events reach frequencies of up to 80%. About half of all editing events depend on ADAR2 while some miRNAs are preferentially edited by ADAR1. Sixty-four percent of all editing events are located within the seed region of mature miRNAs. For the highly edited miR-3099, we experimentally prove retargeting of the edited miRNA to novel 3′ UTRs. We show further that an abundant editing event in miR-497 promotes processing by Drosha of the corresponding pri-miRNA. We also detect reproducible changes in the abundance of specific miRNAs in ADAR2-deficient mice that occur independent of adjacent A to I editing events. This indicates that ADAR2 binding but not editing of miRNA precursors may influence their processing. Correlating with changes in miRNA abundance we find misregulation of putative targets of these miRNAs in the presence or absence of ADAR2.

## INTRODUCTION

Post-transcriptional A to I editing of RNAs is catalyzed by adenosine deaminases that act on RNAs (ADARs). They convert adenosines into inosines in double-stranded RNA structures by hydrolytic deamination. Mammals have three isoforms of ADARs designated as ADAR1 (also known as ADAR), ADAR2 (also known as ADARB1) and ADAR3 (also known as ADARB2). ADAR3, which is predominantly expressed in the brain, is considered to be enzymatically inactive as it has no known editing substrates (1). Besides the catalytic deaminase domain, ADARs have two to three double-stranded RNA binding domains (dsRBDs) by which they bind double-stranded or structured RNAs. dsRBDs show little sequence preference but can specifically position themselves on certain structures such as terminal loops (2,3). Consequently, ADARs can do both, promiscuously edit some perfect double-stranded substrates like inverted repeats and function site specifically, as found in some coding RNA targets (1). As inosine is interpreted as guanosine (G) by most cellular machineries, editing can lead to mRNA recoding (4). Moreover, editing can also alter the stability of the edited RNA or change its secondary structure. In mammals, ADAR1 and ADAR2 are essential proteins. *Adar2^−/−^* knockout mice die 20 days after birth with strong epileptic seizures. This phenotype can be rescued by expression of a pre-edited version of glutamate receptor subunit B (GRIA2, also known as GluA2) suggesting that *Gria2* is the most relevant target of ADAR2 (5,6). ADAR1 deficient mice, in contrast, die embryonically, show a high interferon signature, increased apoptosis and hematopoietic defects (7–9). The exact molecular basis of the ADAR1-dependent lethality is still enigmatic.

Recent screenings of RNA-Seq data have led to the discovery of a large number of editing events (10–14). While some of these editing events affect coding regions of mRNAs, the majority are found in non-coding regions of mRNAs and in non-coding RNAs (10,13,15). Moreover, microRNA (miRNA) precursors are a prominent target for editing by ADARs as their stable stem-loop like structures provide good binding sites for dsRBDs (16–18).

Primary (pri)-miRNAs are typically transcribed by polymerase II and processed in a two-step process. First, the Drosha-DGCR8 microprocessor cleaves pri-miRNAs in the cell nucleus resulting in the production of 60–70-nucleotide-long precursor (pre)-miRNAs. After nuclear export by Exportin-5, the pre-miRNAs are further processed by Dicer-1 to give rise to 20–23-nucleotide-long mature miRNAs. Mature miRNAs are finally incorporated into the RNA-induced silencing complex (RISC), mediating a block in translation or degradation of base complementary mRNAs (19,20). mRNA target recognition mainly occurs via base pairing with the so-called ‘seed sequence’ located between nucleotides 2 and 8 of the mature miRNA (21–23).

Previous work has shown that editing of pri-miRNAs can interfere with their processing by either Drosha or Dicer-1. Moreover, the presence of multiple inosines can also lead to the degradation of miRNA precursors by Tudor-SN (24–26). Editing in the seed sequence of miR-376 can also inhibit their targeting or lead to their retargeting to novel substrates (1,16,27). Interestingly, miRNA processing can be affected by ADARs independent of their A to I editing activity (28). This notion is in agreement with the finding that in the mouse many miRNAs are deregulated in the absence of ADARs even if they appear not edited (29). Similarly, a strong effect of ADARs on miRNA abundance despite low editing levels was observed in *Caenorhabditis elegans*, showing that an editing-independent effect of ADARs on miRNA abundance is conserved from nematodes to mammals (30). These indirect effects of ADARs on miRNA expression may be caused by binding of the double-stranded structures in miRNA precursors. Moreover, ADAR1 was also found to stimulate DICER activity, thereby providing another mechanism that may affect miRNA levels (31).

Previously, we found that the abundance of many miRNAs changes, independent of their editing status in ADAR knockout mouse embryos. Here, ADARB1 (ADAR2) had the strongest effect on miRNA abundance (29). However, in early embryos editing activity on miRNAs and other substrates is generally low but increases during development (18). In the present study, we therefore wanted to determine the impact of ADAR2 on the abundance and sequence of mature miRNAs at a later developmental stage. For this purpose, we applied next-generation sequencing to adult brain of wild-type and *AdarB1^−/−^* mice both carrying the pre-edited *Gria^R/R^* allele that rescues *AdarB1*^−/−^ lethality. Comparison of the miRNA profile of sibling mice of both genotypes allowed us to detect both changes in mature miRNA abundance and A to I editing in mature miRNAs at high sensitivity.

## MATERIALS AND METHODS

### Mice

The *Adar2^−/−^* knockout mouse was a kind gift of Peter Seeburg (5). These transgenic mice are in an SV129 background. As ADAR2 deficiency leads to early postnatal lethality, the mice were rescued with a pre-edited Gria2 receptor (*Gria2^R/R^*) (6). Mice were bred in our facility animal house. *Gria2^R/R^; AdarB1^+/−^* were intercrossed. The resulting sibling female offspring of genotype *Gria2^R/R^; AdarB^−/−^* and *Gria2^R/R^; AdarB1^+/+^* was euthanized at the age of 5.5 months. Whole brain was dissected and subsequently used for RNA preparation from three biological replicates (5,8).

### RNA extraction and miRNA cloning

Female mouse whole brain was dissected at the age of 5.5 month, homogenized and total RNA was extracted using TriFast reagent according to manufacturer's instructions (PEQLAB Biotechnologie GmbH, Erlangen, Germany). miRNA library preparation was performed as previously described (29).

### Sequencing and clipping of reads

Completed libraries were quantified with the Agilent Bioanalyzer dsDNA 1000 assay kit and Agilent QPCR NGS library quantification kit. Cluster generation and sequencing was carried out using the Illumina Genome Analyzer IIx system according to the manufacturer's guidelines. Illumina sequencing was performed at the CSF NGS Unit (csf.ac.at). After sequencing at a read length of 36 base pairs, adaptor sequences were removed using Cutadapt (32).

### Mapping to mature miRNA sequences

Mapping of clipped reads to mature miRNA sequences was performed as described (see Manuscript: Adenosine deaminases that act on RNA induce reproducible changes in abundance and sequence of embryonic miRNAs (29)). Mapping was performed using NextGenMap, restricting the mapped reads to have at least 90% identity (# differences/alignment length) (33).

### Identification of significant editing events

Identification of significant editing events was performed as described (29). Briefly, a χ^2^ test was used to identify putative editing events. Because A-to-G editing events are of interest, the test was applied for each A in the mature miRNA sequences taken from miRBase. The number of expected A's was estimated by the overall perfect match rate over all alignments. The number of expected G's was estimated by the number of observed substitutions (besides A to G) over all alignments. The multiple tests problem was addressed by using Benjamini and Hochberg's method ‘fdr’ (34). Events that were significant in at least two out of three replicates were considered as relevant and used for further analysis.

Identification of T-to-C editing events was done analogously. Here, however, the overall mismatch rate was approximated by the sequencing error rate (0.001). To determine significance levels for editing events, the reads for each miRNA were normalized to the total read counts per sequencing lane, prior to performing statistical analysis.

### Cell culture

U2OS and Hek293 cell lines were grown in Dulbecco's modified Eagle's medium (DMEM) (Lonza) with 10% fetal bovine serum (FBS), 100-U/ml penicillin and 0.1-mg/ml streptomycin. Cells were cultured at 37°C in a 5% CO_2_ incubator with humidified air. Mouse embryonic fibroblasts (MEFs) were isolated from ADAR1^−/−^, ADAR2^−/−^ embryos at day e11.5 and cultured in DMEM supplemented with 20% FBS. A cell line was established by knocking down p53 using plasmid-delivered shRNA (35).

### Reverse trascriptase-quantitative polymerase chain reaction (RT-qPCR)

For the quantification of *Npas4*, total RNA prepared for RNASeq was DNase I digested (Fermentas 1 U/μl, 10 U) and reverse transcribed using M-MLV Reverse Transcriptase (Thermo Scientific) and random hexamers in a total volume of 20 μl. Real-time polymerase chain reaction (PCR) was performed on a Bio-Rad iQ5 mastercycler, using GoTaq SYBR Green qPCR master mix (Promega) and the following primers: *Npas4:* sense 5′-TAAGGGAGGGGAGCAACTTT-3′, antisense 5′-GCCCCTAGGAGTGGAGAACT-3′, *β-actin:* sense 5′-CTTTGCAGCTCCTTCGTTGC-3′, antisense 5′ACGATGGAGGGGAATACAGC 3′; and the PCR program: 40 cycles of 95°C/15 s, 57.5°C/30 s, 72°C/1 min, preceded by denaturation at 95°C/3 min. The relative difference in expression of *Npas4* was calculated by the ΔΔ*C_t_* method using *β-actin* as a reference gene.

### Microarray analysis

Total RNA of two wild-type and two *Adar2^−/−^* was isolated as described and sent for microarray analysis to KFB Regensburg. Samples were prepared using Ambion^®^ WT Expression Kit and analyzed on an Affymetrix GeneChip (Mus musculus).

### Constructs

*Pri-miRNA constructs*: Roughly 120-nucleotide-long sequences of mmu-pri-mir-3099 and -497 were PCR amplified from total mouse DNA and cloned into pSUPERIOR.neo+gfp using the following primers: mir-3099: 5′-GATCAGATCTGAAACCTCAAGCCTGCTGAA-3′ and 5′-GATCAAGCTTAATCCTGCATTCGATGCTCT-3; mir-497: 5′-GATCAGATCTCTCTCGACCCACCCCAGT-3′ and 5′-GATCAAGCTTCATCGGTGCCTCCCATAC-3′. The change from A to G at editing site in mmu-miR-497*, position 20 was accomplished by PCR site-directed mutagenesis. *Targets of miR-3099*: The regions of the 3′ untranslated regions (UTRs) of Vcan and Nap1l1 containing the miRNA binding sites were PCR amplified from total mouse DNA and cloned into the pmirGLO Dual-Luciferase miRNA Target Expression Vector (Promega) using the following primers: Nap1l1: 5′-GATCGAGCTCGATCAGAACCCAGCCGAGT-3′ and 5′-GATCTCTAGAGCATCCCATGAGAGCTAAAACT-3′; Vcan: 5′-GATCGAGCTCGCGCTGATCCTTAAAATGGC-3′ and 5′-GATCTCTAGAATTTACATGGCCATCGGTGC3′.

### Dual luciferase assay

To determine luciferase reporter expression, cells were co-transfected in 24-well plates using JetPEI reagent (Polyplus, Illkirch, France) following the manufacturers instruction. After 6 h of transfection, cells were washed and incubated for 24 h prior to lysis and luciferase measurements using the dual luciferase (renilla versus firefly) assay kit from Promega (Madison, WI, USA). Readings for renilla luciferase were normalized to firefly luciferase (expressed from the same vector (pmiRGlo). Experiments were done in at least three biological replicates always measuring three technical replicates.

### Northern blot

Hek293 cells were transfected using JetPEI reagent (Polyplus, Illkirch, France) following the manufacturer's instruction. After 6 h of transfection, cells were washed and incubated for 24 h prior to RNA extraction using Trifast (peqGOLD *TriFast*, PEQLAB). For northern blot analysis, 10 μg of total RNA were loaded on a 10% PAA gel (8M urea 1x TBE) and separated (for detailed northern blot protocol see (36)). For detection of pri- and pre-miR-497, the following probe: 5′-TTTGGACGTGGCCACAGTGCCG-3′ (complementary to the loop region of the miRNA) was end-labeled using PNK (Thermo Scientific) and γ-ATP[32] according to manufacturer's protocol and hybridized over night at 37°C.

## RESULTS

### ADAR2 reproducibly affects abundance of mature miRNAs in the brain

To determine A to I editing but also changes in relative abundance induced by ADAR2, we isolated small RNAs (19–25 nucleotides) from adult *Gria2*^R/R^ and *Adar2^−/−^*, *Gria2*^R/R^ female mouse brains and subjected them to deep sequencing (The *Adar2^−/−^*, Gria2^R/R^ mouse strain was a kind gift of the lab of Peter Seeburg, MPI, Heidelberg (6,8)). We chose to use whole adult brain for our analysis, as editing levels are known to be very high in the nervous system (27). Different genotypes were picked from siblings from heterozygous crosses. For each genotype and biological replicate one lane on an Illumina Genome Analyzer II was used. On average, 29 million reads were obtained per lane. Of these about 71% were mappable to annotated mature miRNA sequences (release 16 of miRBase (37), see the Materials and Methods section). Reads were mapped with NextGenMap (33) using a 90% identity threshold (roughly two mismatches per read) to detect potential editing events. Three independent biological replicates of each genotype were examined for all analyses. After mapping, reads were normalized to total mapped miRNA reads per sample. Of the 1055 mature miRNAs annotated in miRBase (Release 16) on average 605 miRNAs were covered by at least 10 reads in all three biological replicates, allowing a maximum of two mismatches.

Interestingly, only a very small set of five miRNAs accounted for 48% of the mappable reads (Supplementary Figure S1A). This group of miRNAs consisted of the same members in all replicates and in both genotypes with mmu-miR-378a being most abundant. None of the five most abundant miRNAs showed a significant difference in abundance in the ADAR2-deficient versus wild-type brains (Supplementary Figure S1B and C). Mmu-miR-378a had already been reported by others to be edited (18,38). This miRNA has been shown to promote cell migration in non-small cell lung cancer brain metastasis (39). Also, in a previous study we detected very low editing of this mmu-miR-378a at embryonic day 11.5 (29). In the adult brain, we find mmu-miR-378a edited to 6.2% at position 16 (Table 4), verifying that editing efficiency of this miRNA increases during development. The editing position at nucleotide 16 is 6 nucleotides away from the DROSHA cleavage site and therefore this editing event is unlikely to interfere with DROSHA activity. The high abundance of mmu-miR-378a in the brain was surprising as this miRNA has not been reported to be highly expressed in the nervous system. However, we detected high expression levels in three independent replicates. No editing could be found for the other four most abundant miRNAs.

**Table 1.** Top 10 of consistently up- or downregulated mature miRNAs

Ten most downregulated miRNAs				Ten most upregulated miRNAs			
mmu-miRNA	Fold down in *Adar2*^−/−^	*P*-value	Avg. read number/RPM	mmu-miRNA	Fold up in *Adar2*^−/−^	*P*-value	Avg. read number/RPM
miR-497*	−2.59	0.0034	2173/104	miR-335-5p	2.76	0.0841	16070/772
miR-1247*	−1.59	0.0027	155/7	miR-15b	2.46	0.0941	124/6
miR-1298*	−1.55	0.0492	3463/166	miR-98	2.27	0.0986	1911/92
miR-495*	−1.54	0.0286	267/13	miR-467a*	2.07	0.0311	266/13
miR-99b	−1.54	0.031	167587/8053	miR-872*	2.01	0.0831	160/8
miR-126-5p	−1.48	0.0604	85213/4095	miR-467d*	1.92	0.0364	1033/50
miR-128-1*	−1.44	0.0023	1636/79	miR-540-5p	1.9	0.0475	887/43
miR-376a*	−1.44	0.0707	7035/338	miR-337-3p	1.89	0.0178	139/7
miR-129-5p	−1.43	0.0805	25055/1204	miR-3107	1.88	0.0178	1657/80
miR-1937a	−1.42	0.0257	223/11	miR-486	1.88	0.0178	1657/80

Only miRNAs with a minimum of 1.3-fold up-or downregulation were considered (Students *t*-test: *P* < 0.1). Underlined miRNAs were found edited in this study. A complete list of up- or downregulated miRNAs can be found in Supplementary Table S1.

**Table 2.** Edited miRNAs showing consistent up- or downregulation

Edited miRNA	Abundance fold change in *Adar2^−/−^*	*P*-value	Avg. read number/RPM	% editing in WT	Editing position
miR-497*	−2.59	0.0034	2173/104	73.2	20
miR-376a*	−1.44	0.0707	7035/338	58.5	4
miR-99b*	−1.39	0.0201	4294/206	15.4	3
miR-151-3p	−1.31	0.0548	189662/9113	2.8	3
miR-873	1.3	0.0876	1155/55	1.3	3
miR-467c	1.32	0.0989	209/10	48.2	3
miR-669c	1.45	0.0094	248/12	3.9	3
miR-467a	1.56	0.0796	874/42	9.2	3
miR-376b	1.72	0.0806	735/35	45.4	6
miR-467d	1.83	0.0546	3711/178	1.1/15.2	2/3
miR-467e	1.83	0.0435	256/12	2.1/26.3	3/4
miR-540-5p	1.9	0.0475	887/43	12.1/2.4	3/9
miR-467d*	1.92	0.0364	1033/50	0.8/4.8	5/9
miR-98	2.27	0.0986	1911/92	1.2/0.6	11/17
miR-335-5p	2.76	0.0841	16070/772	1.5	4

Only edited miRNAs with a minimum of 1.3-fold up-or downregulation were considered (Students *t*-test: *P* < 0.1).

**Table 3.** Significantly deregulated mRNAs in *Adar2^−/−^* brain (*P*-value <0.05)

Name	Fold change	*P*-value	Description
Prl	17.3	0.03682	Prolactin
Snora16a	2.8	0.03165	Small nucleolar RNA, H/ACA box 16A
snoRNA	2.7	0.00763	Mus musculus clone MBI-31 H/ACA box snoRNA, partial sequence
snRNA	2.6	0.00732	ncrna: snRNA chromosome:NCBIM37:15:89884840:89884946:-1
Snora17	2.5	0.02595	Small nucleolar RNA, H/ACA box 17
Name	Fold change	*P*-value	Description
Adarb1	−2.7	0.00557	Adenosine deaminase, RNA-specific, B1
Serpinb1a	−2.0	0.01642	Serine (or cysteine) peptidase inhibitor, clade B, member 1a
Mir9-2	−2.0	0.01149	MicroRNA 9-2
Npas4	−2.0	0.01874	Neuronal PAS domain protein 4

Results of a whole genome microarray analysis of wild-type and *Adar2*^−/−^ mouse brain. Only deregulated mRNAs with a *P*-value <0.05 are shown.

**Table 4.**
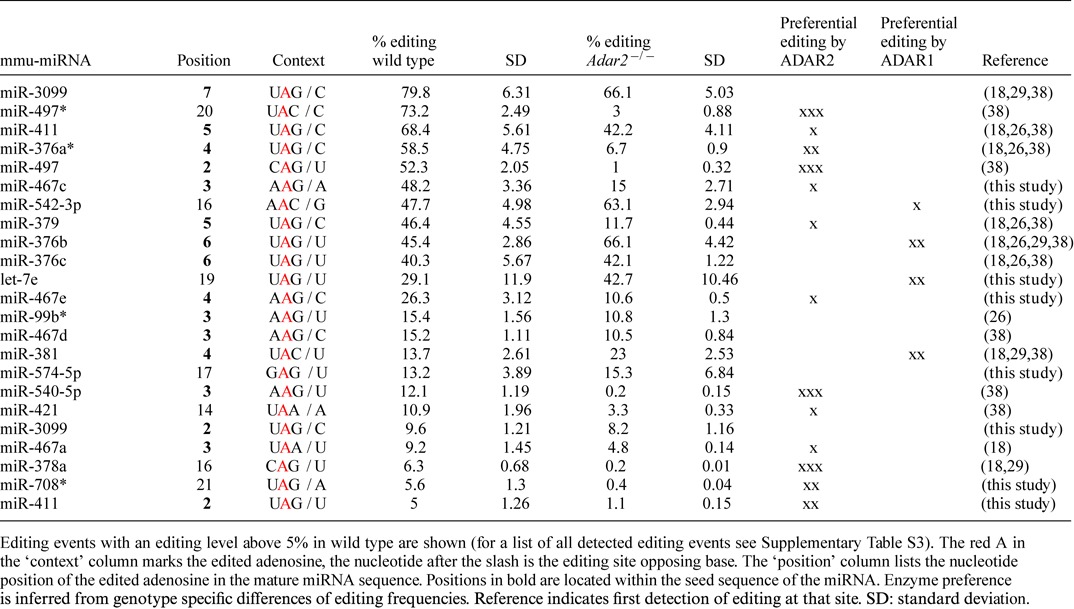
Significant editing events detected in miRNAs in all three replicates

**Figure 1.**
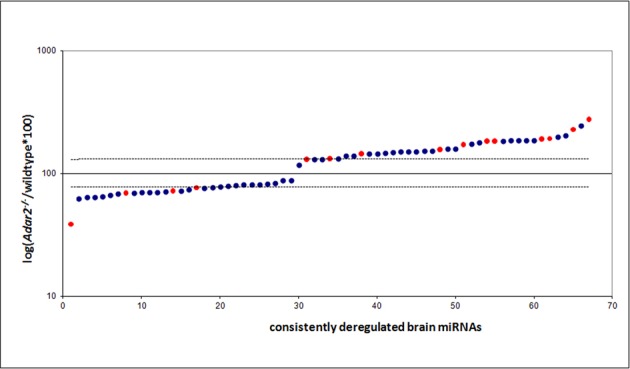
miRNAs are consistently up- or downregulated in *Adar2^−/−^* mice, independent of their editing status. miRNAs with a read coverage greater than 100 reads were sorted according to their relative abundance to wild type (displayed as% of wild-type abundance) in the ADAR2 knockout genotype and plotted on a semi-logarithmic scale. In blue and red are unedited and edited miRNAs, respectively. Wild-type expression level is represented by 100, and a threshold of 1.3-fold deregulation is marked by thin lines. miRNAs with relative changes in abundance smaller than 1.15-fold are not displayed. For a complete list of all consistently deregulated miRNAs see Table 1 and Supplementary Table S1.

The subsequent analysis of changes in miRNA abundance between wild-type and ADAR2 knockout mice was restricted to the 455 miRNAs that are at least covered by 100 reads in each sequencing replicate, to stay biologically relevant. From these miRNAs 67 showed a consistent change in abundance in brains of *Adar2^−/−^*-deficient animals in all three replicates. Of these, 17 miRNAs (26%) were downregulated and 37 miRNAs (56%) upregulated by at least 1.3-fold (Figure 1). The remaining 13 miRNAs with changes smaller than 1.3-fold were not considered further. Thus, two times more mature miRNAs show upregulation than downregulation in *Adar2^−/−^* brains. The highest change in abundance was 2.76-fold up for miR-335-5p and −2.59-fold down for miR-497* (Table 1).

Thus, in total we find ∼8% of all miRNAs expressed in the adult brain to be upregulated in the absence of ADAR2. A complete list of up- and downregulated miRNAs in the brain is shown in Supplementary Table S1.

It has been reported for some miRNAs that editing near a processing site can lead to a change in processing efficiency of the miRNA (24–26). Thus, if editing interferes with processing, one would expect a change in abundance of the mature miRNA in the absence of the editing enzyme. Along these lines, we also find edited mature miRNAs among the deregulated miRNAs (Table 2). However, similar to the data from E11.5 embryos also many unedited miRNAs are up- or downregulated in the absence of ADAR2, while many edited miRNAs are not consistently deregulated (compare Figure 1 and Tables 1 and 2) (29).

Of the 59 miRNAs that we found edited in our study, only 15 were consistently up- or downregulated by at least 1.3-fold (four miRNAs downregulated, 11 miRNAs upregulated). Additionally, the three most upregulated edited miRNAs show only low editing efficiencies (0.8–4.8%) in wild type (Table 2). Together, this argues for an additional editing-independent effect of ADAR2 on processing of miRNAs in the adult brain, most likely by ADAR2 binding to miRNA precursors. Similar findings were made for miRNAs expressed in mouse embryos (29) and in *C. elegans* (30).

Next, we analyzed overall changes in mRNA levels between wild-type and *Adar2^−/−^* brains using a mouse genome microarray (Affymetrix GeneChip mouse). This analysis showed five significantly upregulated (*P*-value <0.05) and four downregulated genes in the *Adar2^−/−^* mice (Table 3). Expecting a correlation of the observed deregulated miRNAs and their targets, we looked for overlaps of predicted targets of the most deregulated miRNAs in the ADAR2 mutant within this small set of deregulated mRNAs. We found *Npas4* (downregulated 2-fold) to be a predicted target of the most upregulated miRNA miR-335-5p (2.76-fold upregulation in *Adar2^−/−^* brain). This mRNA encodes the Neuronal Per Arnt Sim (PAS) Domain Protein 4, NPAS4, a neuron-specific, basic helix–loop–helix transcription factor that is implicated in hippocampal neurogenesis (40). In order to validate the result of the microarray analysis, we independently measured *Npas4* mRNA levels by RT-qPCR on brains of wild-type and ADAR2-deficient mice. The RT-qPCR showed a 2.54 ± 0.41-fold downregulation of *Npas4* in *Adar2^−/−^* brains (Supplementary Figure S2; *n* = 3).

### A to G transitions drop significantly in the absence of ADAR2

A lack of ADAR2 should lead to a decreased number of A to G changes, which reflect A to I editing, in mature miRNAs. However, as the ADAR1 enzyme is still present and active in *Adar2^−/−^* mice, residual editing by ADAR1 would still be expected. Thus, we analyzed the relative frequency of A to G transitions compared to all other 11 possible nucleotide transition types in wild-type and ADAR2 knockout brains.

**Figure 2.**
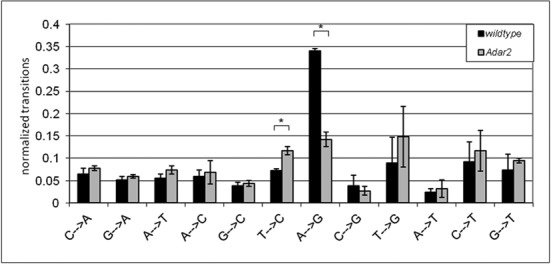
Relative A to G transitions drop significantly in mice deficient in ADAR2. All 12 possible nucleotide transitions in the mapped miRNA reads from wild-type and *Adar2^−/−^* mice were summed up and normalized to total transition numbers in all three replicates. * Students *t*-test *P* < 0.002.

We analyzed mapped reads with maximally two mismatches so that we were able to detect deviations from the annotated miRNA sequences. We also discarded reads that mapped to the reverse complement of the mature miRNAs, as our cloning protocol for mature miRNAs is strand-specific. During mapping, weighting was applied when there were several miRNAs with equally good alignment scores. We considered trimming of the last few nucleotides of the reads as unnecessary, because NextGenMap (33) computes a local optimal pairwise sequence alignment (41), which by itself ignores mismatches toward the very end of the read. We recorded the number (0–2) and type of mismatches for each read and each position in the mapped read. By doing so a count table of all mismatch types for both genotypes and all three replicates was generated. The occurrence of each mismatch type was normalized by the sum of all mismatches in each biological replicate. This way, we were able to compare the general mismatch profile between wild-type and *Adar2^−/−^* miRNAs. Although, we detected all 12 possible mismatch types in the mapped reads, the relative frequency of A to G mismatches was the most prevalent one in wild type (Figure 2). The number of A to G transitions dropped significantly in the *Adar2^−/−^* brain to 41% of wild-type level (Students *t*-test *P* < 3.95E−05). This strong effect of ADAR2 on the overall A to G transition level of miRNAs demonstrates that ADAR1 only weakly contributes to the observed editing events.

Also a second mismatch, namely T to C (which reflects a U to C change in the RNA sequence), showed a genotype-specific difference (Figure 2). The number of T to C transitions was increased by 38% in the ADAR2-deficient background (Students *t*-test *P* < 1.45E−03). However, when we tested for individual, significant T to C events at specific locations that were present in *Adar2^−/−^* but not in wild type (see the Materials and Methods section), no single miRNA was detected that showed a significant T to C transition frequency above 1.9%. Overall, only 15 miRNAs showed T to C transitions exclusively in the ADAR2 knockout, most of them with a frequency lower than 0.5% (Supplementary Table S2). Of these 15 miRNAs only mmu-let-7f showed an additional significant A to G change, albeit with a similarly low frequency of 0.42%. It thus appears that T to C transitions are distributed stochastically in the absence of ADAR2.

The other 10 mismatch types showed no genotype or position specificity and thus were considered as results from sequencing and sample preparation errors.

### High editing levels of mature brain miRNAs influence miRNA processing or targeting

It is known that editing levels in mRNAs and miRNAs increase during pre- and post-natal development (18,42). Thus, we expected a moderate to high editing frequency of mature miRNAs in adult mouse brains. To determine editing levels of mature miRNAs in wild type, and to reveal possible target specificity of ADAR2 versus ADAR1, we analyzed our data for significant editing events in all miRNAs in the two genotypes. In each biological replicate A to G transitions were counted for individual positions in all miRNAs from wild-type and *Adar2^−/−^* brains. Using a chi-squared test (see (29)) an A to G change was assumed significant when the observed number of Gs at that position was higher than expected due to sequencing errors and experimental noise. Only events for which a significant A to G transition was observed in all three replicates were considered for further analysis. Again, only miRNAs covered by at least 100 reads were considered. Doing this, we ended up with a list of 72 significant editing events in 59 miRNAs (Table 4; complete list in Supplementary Table S3).

The highest editing level is found in mmu-miR-3099 at position 7 of the mature sequence, which is edited to 80% in wild type, and to 66% in ADAR2 mutant brain (Table 4). miR-3099 has already been found to be edited at this position in other studies (18,38,43).

Editing in the seed sequence can lead to retargeting of the miRNA, which was already demonstrated for miR-376a-5p (former miR-376a*) (27). As miR-3099 is edited up to 80% in the brain, the major amount of this miRNA has the potential to target a different set of mRNAs. Accordingly, the TargetScan Custom algorithm (http://www.targetscan.org/vert_50/seedmatch.html, using default settings) predicts a set of 58 targets for unedited miR-3099, whereas 161 targets are predicted for the edited version, with only three mRNA targets present in both sets.

**Figure 3.**
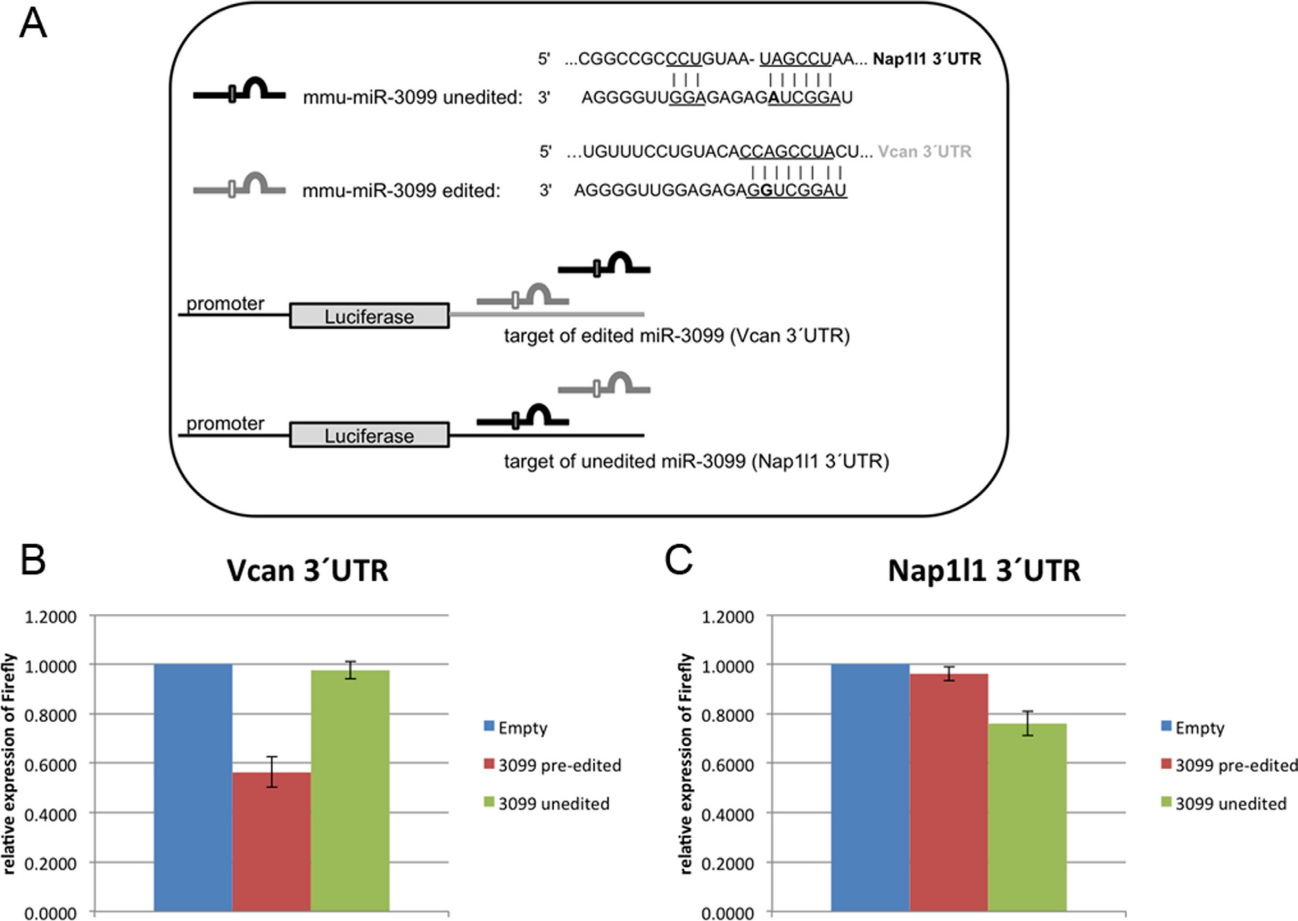
Editing of miR-3099 changes its target specificity. (**A**) The 3′ UTRs of *Nap1l1*—target of mmu-miR-3099—and *Vcan*—target of edited mmu-miR-3099—were introduced into pMirGlo downstream of the firefly luciferase open reading frame (ORF). The same plasmid also expresses Renilla luciferase as a reference. (**B**) Relative luciferase activity for the two reporter constructs was measured for the *Vcan* and (**C**) *Nap1l1* harboring plasmids 24 h after cotransfection with control (blue), edited (red) or unedited (green) pri-miR-3099 (*n* = 3). The *Vcan* 3′UTR responds to edited miR-3099 while the *Nap1l1* UTR shows a stronger response to unedited miR-3099.

As miR-3099 is edited by the essential ADAR1 enzyme (see below) we could not test for changes in the expression of predicted targets in the absence of ADAR1 *in vivo*. Still, to test for a change of target specificity *in vivo* we selected the best target predictions for unedited and edited miR-3099, *Nap1l1* and *Vcan*, respectively and cloned their 3′UTRs into the pmiRGlo vector for analysis by dual luciferase assay. Both, the *Nap1l1* and *Vcan* UTRs contain at least one miRNA binding site for the respective miRNA that is conserved from chicken to human. We co-transfected the two different reporter constructs (pmiRGlo + *Nap1l1* or *Vcan* 3′UTR) with either unedited or pre-edited pri-miR-3099 or empty vector control (pSuperior-neo-GFP) into human HeK293 cells and measured the effect of the two different miRNA versions on protein expression of the reporters. The *Vcan* 3′ UTR, the predicted target of edited miR-3099, showed a 44% reduction compared to empty vector control when cotransfected with edited miR-3099 but not with the unedited version. Also the *Nap1l1* 3′ UTR led to a 24% reduction in the presence of the unedited miRNA but not with the edited form (Figure 3). Transfection efficiencies tested by GFP expression from the miRNA expressing pSuperior constructs were ∼10% for both, edited and unedited miRNA.

HeK293 cells show little or no detectable ADAR2 activity. Still ADAR1 activity had been detected in these cells (44). Therefore, to exclude the possibility that the reporter assay was altered by editing of the transfected pri-miR3099 by ADAR1, the above-mentioned experiments were repeated in immortalized MEFs derived from *Adar1^−/−^*, *Adar2^−/−^* embryos (Supplementary Figure S3). These cells are entirely devoid of all ADAR activity (Vesely, C. and Jantsch, M.F.; personal communication). Still, cotransfection of pre-edited miR-3099 led to an ∼22% reduction of the *Vcan* reporter while the unedited miR-3099 did not affect reporter gene expression. In contrast, unedited miR-3099 reduced *Nap1|1* reporter gene expression by 30% while the edited version had only reduced gene expression by 14%.

Taken together these results demonstrate that editing in the seed region strongly redirects miR-3099 from one target to another.

*Vcan* encodes the proteoglycan Versican that stimulates angiogenesis and can promote tumor growth. Consistently, *Vcan* is expressed well during embryogenesis but not in adult tissues. Age- and differentiation-dependent editing of miR-3099 may well be a mechanism repressing *Vcan* expression in adult, differentiated tissues (45).

miR-3099 is upregulated during neuro-differentiation and might therefore also play a role in embryogenesis and neuronal function (46). Using GeneCodis modular and singular enrichment analysis (http://genecodis.cnb.csic.es, (47–49)) we looked for enrichment in KEGG pathways in the two sets of predicted targets of miR-3099. The genes potentially targeted by the edited form were enriched for (among others): ‘Glutamatergic synapse’ (five genes), ‘Axon guidance’ (four genes) and ‘Notch signaling pathway’ (three genes), whereas the targets of unedited miR-3099 were not (Supplementary Figure S4). This would be in agreement with a possible requirement of editing of miR-3099 for its proper function in neuro-differentiation.

**Figure 4.**
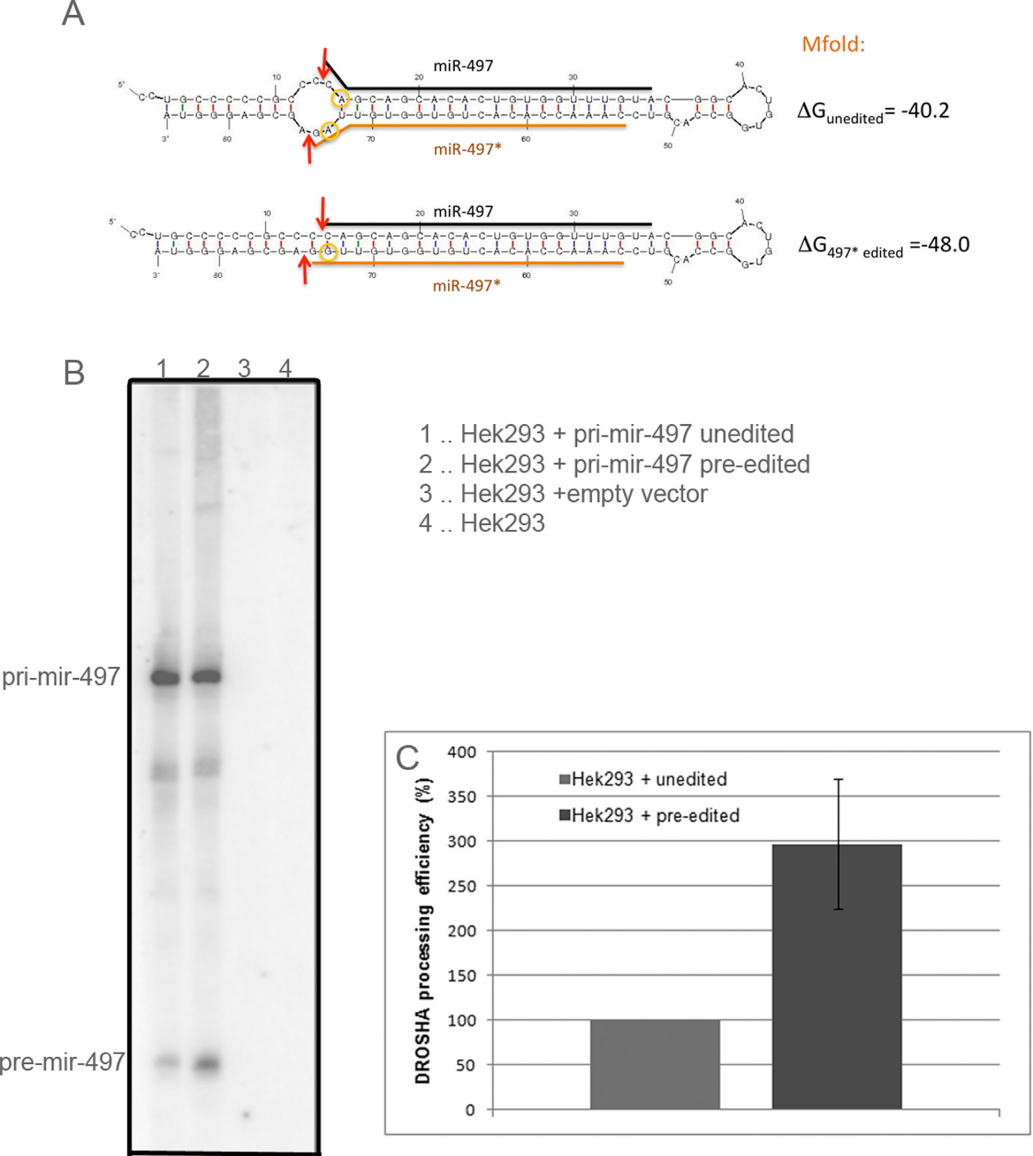
Editing of pri-mir-497 enhances its processing efficiency. (**A**) Structure prediction by mfold (http://mfold.rna.albany.edu) for unedited (top) and edited pri-miR-497 (editing at position 74 = position 20 in miR-497*; bottom). Editing sites are marked by yellow circles. Mfold energy predictions of unedited and singly edited pri-miR-497* are indicated. The orange and black straight lines symbolize the mature miRNA sequences while arrows mark the DROSHA cleavage sites. (**B**) Representative northern blot of HEK293 cells transfected with pSuperior expressing unedited (1), pre-edited (2) or no miRNA (3). Processing of pri- to pre-miRNA was analyzed by hybridization with an antisense oligonucleotide. (**C**) Quantification of processing efficiency (ratio pre-miR/pri-miR) from three experiments.

The second most edited miRNA was mmu-miR-497* (mmu-miR-497-3p), which is edited to 73% at position 20 in wild type. Interestingly, editing is almost lost in the absence of ADAR2 as the efficiency drops to only 3% in the knockout, indicating that this site is edited almost exclusively by ADAR2. Also mmu-miR-497 is found highly edited (52%) in wild type and almost unedited in *Adar2^−/−^* (1%). The editing sites, position 20 in miR-497* and position 2 in miR-497, are opposite each other in the double-stranded pre-miRNA structure (Figure 4A), suggesting that ADAR2 can edit both sides of the stem loop. However, as we only analyzed mature sequences and not pre-miRNAs we do not know whether the two editing events occur simultaneously or on different molecules. Still given the high frequency of both editing events of 73% and 52%, respectively, some precursor molecules must be edited at both sites simultaneously. Whether this is done by the same ADAR2 molecule or in successive editing reactions is not clear from our data.

When pre-mir-497 is edited at position 20 of its star sequence, the precursor stem loop gets stabilized (Δ*G*_unedited_ = −40.2 kcal/mol, Δ*G*_497*edited_ = −48.0 kcal/mol, calculated by mfold, http://mfold.rna.albany.edu, using default setting, (50)). Editing at position 2 of miR-497 does not change RNA stability compared to the unedited stem loop, and the fully edited version of the pre-miRNA is only slightly less stable than the single editing in 497* alone (Δ*G*_fully edited_ = −47.2 kcal/mol) (Figure 4A). Thus, editing of position 20 of miR-497* changes the structure around the DROSHA cleavage site and thereby might enhance DROSHA cleavage efficiency. Confirming this hypothesis, we found miR-497* as the strongest downregulated miRNA (−2.6-fold) in ADAR2-deficient brain (see Tables 1 and 2).

To demonstrate the role of editing on pri-mir-497 processing more directly we analyzed the efficiency of the DROSHA cleavage step using Hek293 cells. Wild-type Hek293 cells that show negligible ADAR2 activity were transfected with either unedited or pre-edited (A to G change at position 20 in mmu-miR-497*) primary miRNA and processing efficiencies from pri- to pre-miRNA were analyzed by northern blots (Figure 4B). The northern blot shows a 3-fold (+/−20%) more efficient processing of edited over unedited pri-mir-497 (Figure 4C). This demonstrates that editing of pri-mir-497 by ADAR2 stimulates processing of the primary transcript. pri-miR-497 is edited to 73% in the brain (Table 2). Thus, the 3-fold increase in processing of edited over unedited primiR-497 correlates well with the −2.6-fold decrease of the mature miRNA level in the brain upon loss of ADAR2.

Mature miR-497 is edited to 52% at position 2 within its seed region. TargetSpy (www.targetspy.org) predicts the SerpinB1a mRNA to be a strong target (score = 0.995) of the unedited but not the edited miRNA. Consistently, we find SerbinB1 mRNA to be downregulated by ∼2-fold in the absence of ADAR2 supporting the idea that this mRNA is targeted by unedited miR-497 (Table 3). This finding is even more astonishing when considering that maturation of miR-497 is downregulated upon lack of editing. SerpinB1 is a member of the Serpin family of proteinase inhibitors that protect cells from inflammatory signaling but also regulates cell migration and invasion in gliomas (51). Moreover, as it was shown that miR-497 regulates neuronal death in mouse brain (52,53), editing of this miRNA may play an important role in fine tuning the effect of miR-497 on apoptosis in neuronal cells.

### Half of edited miRNAs in the adult brain are preferentially edited by ADAR2

To determine editing preferences of the identified sites for ADAR2 or ADAR1, editing levels were also calculated for miRNAs obtained from ADAR2-deficient mouse brains. Interestingly, the editing levels of many miRNAs dropped significantly in the mutant mice. Fifty-one percent of all editing events detected in wild type showed a reduction of editing levels by 50% in *Adar2^−/−^* brains. In 26% of all sites editing was no longer detectable in the absence of ADAR2 (Table 4 and Supplementary Table S3). Also, the six most highly edited miRNAs show a reduction in editing efficiency in the absence of ADAR2. However, in some cases the absence of ADAR2 obviously stimulated editing by ADAR1. Editing levels increased by at least 50% in 11% of all editing events (eight positions in seven miRNAs): miR-376b (position 6), let-7e (positions 17 and 19), miR-381 (position 4), miR-467d* (position 9), miR-3061-3p (position 3), miR-151-3p (position 3), miR-219-3p (position 15). This suggests that binding by ADAR2 can compete for editing by ADAR1 on some miRNA precursors. The data also indicate that ADAR2 and ADAR1 have different preferred pri-miRNA targets and that ADAR2 plays an important role in miRNA editing in the brain.

### Enrichment of editing in the seed region of mature miRNAs

**Figure 5.**
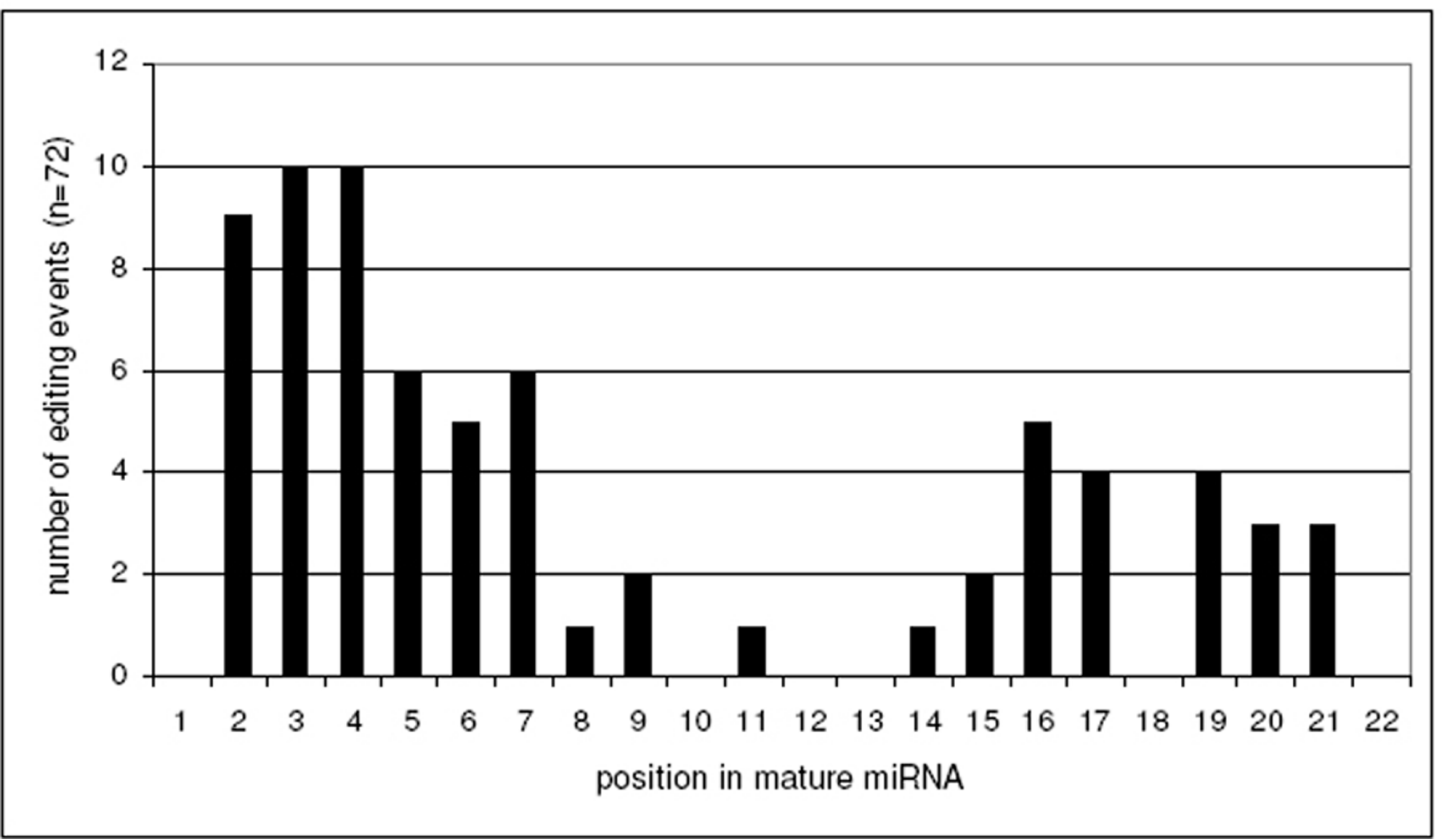
Editing events are enriched in the seed sequence of mature miRNAs. The frequencies of all detected editing events within the mature miRNAs (22 nts average length) are plotted in absolute numbers. The distribution of editing events shows a clear preference for the nucleotides 2–7, all located within the seed region.

When examining the positions that showed significant editing events in mature miRNAs, we found that 64% of all editing events occur at position 2–7, namely the seed sequence (Figure 5). Only 46% of all editing events occur in the remaining 16 positions, if an average length of 22 nucleotides is assumed. This enrichment of editing in the seed sequence shows that ADARs have the potential to play an important role in the modulation of miRNA target specificity in the brain.

Looking at the context around editing sites in mature miRNAs we found that a U>A>C>G upstream of the edited A is favored, and downstream ADARs prefer a G>C≈A>U. The nucleotide opposing the editing site is usually either a C or a U and rarely an A or a G (Supplementary Figure S5). This is in concordance with known editing site preferences (54,55).

### New editing sites in mature miRNAs

Of the 72 detected editing events only one third (24) had already been published earlier (Supplementary Table S3). Of these, the majority (65%) showed editing levels of more than 5% (see Table 4). Eight newly identified editing events show editing levels of at least 5%. Four of these abundant newly identified events harbor the edited adenosine in the seed region (miR-467c, position 3; miR-467e, position 4; miR-3099, position 2; miR-411, position 2).

Three of the newly identified editing sites show higher editing levels in the absence of ADAR2 (miR-542-3p, position 16; let-7e, position 19; and miR-574-5p, position 17), showing that these miRNAs are preferentially edited by ADAR1 (Table 4). Increased editing in the absence of ADAR2 also suggests that editing by ADAR1 might be blocked by ADAR2 binding, without editing them. In contrast, editing sites in all other newly identified edited miRNAs, except for position 2 of miR-3099, are significantly less edited in the ADAR2 mutant. This shows that they are preferentially edited by ADAR2 (miR-467c at position 3, miR-467e at position 4, miR-708* at position 21 and miR-411 at position 2) (see Table 4).

## DISCUSSION

In this study, we have determined changes in sequence and abundance of mature miRNAs in wild type versus ADAR2 (also known as ADARB1) deficient adult mouse brains. We demonstrate that clear and reproducible changes in the abundance of a set of miRNAs occur in the absence of ADAR2. Variations range from a 2.6-fold downregulation to a 2.8-fold upregulation in the absence of ADAR2. In general, however, loss of ADAR2 leads to a significantly larger proportion of upregulated miRNAs. Similar to previous studies in mouse embryos and in *C. elegans*, deregulation of miRNAs seems independent of RNA editing in the adult brain (29,30). This is documented by the fact that both unedited miRNAs are found deregulated in the absence of ADAR2, while some highly edited miRNAs show no changes in abundance at all. This finding is in agreement with the idea that binding of ADARs to miRNA precursors or interaction of ADARs with miRNA processing factors can influence miRNA processing without editing. It should be noted, however, that by sequencing mature miRNAs, editing events that are located only in the precursor, outside the mature miRNA, are not detected in our study. Still, as editing events that affect processing should lie within the immediate vicinity of the DROSHA or DICER cleavage sites, it is highly unlikely that all misregulated miRNAs can be attributed to such an event. Moreover, ADARs may also regulate miRNA processing by affecting the expression or activity of components of the miRNA processing machinery. A similar indirect effect was recently described for the impact of ADARs on the control of alternative splicing (56).

As miRNAs are important regulators of mRNA translation we can observe reproducible changes in mRNA expression profiles in the absence of ADAR2. For instance, we can detect a strong downregulation of *Npas4* mRNA correlating with miR-335-5p upregulation. This finding nicely underlines the biological relevance of ADAR2-dependent changes in miRNA abundance.

Compared to our previous study performed in mouse embryos, a smaller number of miRNAs are found to be consistently deregulated in the absence of ADAR2 in the adult brain (29). As expected, however, we find a larger number of miRNAs edited to a much higher extent in the adult brain than at the early embryo. This is in agreement with published data showing that miRNA editing drastically increases during development (18). Thus, in contrast to embryos where ADARs mainly affect miRNA abundance, in the adult brain some miRNAs are strongly affected by the actual editing reaction leading to changes in target specificity. As we only consider ADAR2 in this study, it is possible that even more mature miRNAs are affected in an adult ADAR1-deficient brain (8).

Currently it is not clear why ADARs interfere more strongly with miRNA abundance but show little editing activity in the early embryo. It is possible, however, that miRNA processing becomes more robust in later developmental stages, therefore becoming less sensitive to competing double-stranded RNA binding proteins.

Interestingly, editing levels in the 5.5-months-old mice used here were comparable to those previously reported for 21-days-old mice (18). This suggests that most miRNA editing events reach a plateau upon reaching adulthood.

A recent study showed that ADAR1 and DICER directly interact and form a more effective miRNA processing complex (31). In this study, a global downregulation of mature miRNAs in ADAR1 single mutant embryos was observed which was explained by the lack of ADAR1–DICER processing complexes (31). Neither in our previous study in mouse embryos (29) nor in the current study in the adult brain could we observe such a global downregulation of mature miRNAs in the absence of ADAR2 or both ADAR1 and ADAR2. In contrast, we find more mature miRNAs upregulated relative to wild type in the knockout mice. These apparently conflicting results might be explained by differences in the temporal regulation of miRNA expression during development, where time differences of 0.5–1 days can be important. In our study performed in embryos the used ADAR1 knockout mice survive in general at least 0.5 days longer than the strain used by Ota *et al.* (31), thus possibly explaining the observed differences (29). Also, as ADAR2 seemingly does not interact with DICER, loss of ADAR2 may only indirectly affect pre-miRNA processing, possibly via competitive binding.

Our study shows that the overall number of A to G changes drops almost by 60% in the absence of ADAR2, although ADAR1 is still present. Some editing events seem to exclusively depend on the presence of ADAR2, while others are not affected by this enzyme. This indicates that ADAR2 plays an important role in introducing A to I changes into mature miRNAs and that individual ADARs must display specificity for certain miRNA targets. Given that the overall structure of pri- and pre-miRNAs is relatively similar, this finding is rather surprising. Apparently, enzyme preferences for certain sites in miRNAs are caused by a combination of subtle differences in the substrates.

We find that editing levels in the adult mouse brain can reach 80%. Thirteen miRNAs show editing levels above 10% within their seed region, which will significantly influence their target specificity. Using a reporter assay we demonstrate that two predicted targets for edited and unedited versions of the most highly edited miRNA, miR-3099, are only affected by the edited and unedited miR-3099, respectively. Interestingly, the two sets of predicted targets for the unedited and edited miRNA isoforms only overlap by three mRNAs. Together this indicates that this particular editing event may lead to dramatic retargeting of miR-3099 in wild-type mice. miR-3099 was identified in a deep sequencing study of the developing mouse brain (46). Due to its spatiotemporal expression pattern it was suggested to possibly influence neural differentiation or function.

Another highly edited miRNA, miR-497, that is edited in the seed region and the star sequence to 52% and 73%, respectively, is not only redirected but also downregulated by more than 2.5-fold, upon loss of editing. As we only analyzed mature miRNAs by sequences we cannot state whether both editing events are linked to each other. However, given the high frequency of both editing events at least 30% of all molecules must harbor both editing events. For miR-497, we could show that a pre-edited version is processed more efficiently by DROSHA. Thus, this miRNA is tightly controlled by ADAR2 by both upregulating its processing and redirecting its target specificity. Interestingly, miR-497 has been found to positively regulate neuronal death in mouse brain and target genes like *Bcl-2*, *Bcl-w*, *cyclin D2* and *SerpinB1* therefore underlining the importance for a tight regulation of this miRNA (52,53). A similar effect of ADAR editing on processing of polycistronic pri-miRNAs has recently been shown in *Drosophila*, where editing can promote but also inhibit the processing of certain miRNAs (57). In the absence of ADAR2, we found decreased *SerpinB1* mRNA levels which is predicted to be targeted by the native but not the edited miR-497 underscoring the impact of RNA editing in the seed region of this miRNA.

Several new editing sites were identified in miRNAs of which four occur in the seed sequence with at least 5% abundance. Of these, miR-467 is edited to 48% in wild type, and hence almost half of the produced miRNAs have the potential to target different mRNAs in wild-type mice. Moreover, besides confirming already published editing sites in miRNAs we could also distinguish editing site preferences of the two enzymes ADAR1 and ADAR2. For example miR-497 is exclusively targeted by ADAR2, whereas miR-376b is edited by ADAR1, as it is even more efficiently edited in the absence of ADAR2. The determined target specificities of ADARs for the edited brain miRNAs will be a valuable basis for future studies on the functional implications of edited miRNAs.

We also detected a global but significant change of T to C transitions in miRNAs isolated from *Adar2^−/−^* mice (possibly representing U to C changes in the RNA). However, those changes were stochastically distributed along the length of all miRNAs and not enriched at a specific position. This finding is in good agreement with other studies that have observed similar low-level T to C changes in RNA-Seq experiments (58,59). Nonetheless, to understand the occurrence and possible site specificity of these events would require deeper sequencing. Thus, at present, the biological relevance of T to C transitions remains obscure. One possible influence of ADARs on the frequency of T to C transitions could come from editing of the mRNA of DNA repair enzymes and thereby changing their enzymatic properties, like it has been shown for NEIL1 (60). Still, further studies are needed to understand the T to C changes and the involvement of ADARs in this transition type.

In summary, our study clearly demonstrates that ADAR2 influences miRNA abundance and targeting at several levels. First, binding of ADAR2 seemingly interferes with miRNA processing, possibly by competitive binding. Second, editing events near Drosha and Dicer cleavage sites can modulate the processing efficiencies of the cognate enzymes. Third, editing in the seed region of mature brain miRNAs can lead to retargeting and therefore diversify miRNA functions in the mammalian brain. Lastly, ADARs may indirectly influence miRNA processing by controlling the expression or activity of other factors involved in the miRNA processing pathway.

## AVAILABILITY

Illumina reads for 21–23-nt-long RNAs for wild-type and *Adarb1^−/−^*-deficient mice and microarray results are available at http://www.ncbi.nlm.nih.gov/geo/.

## ACCESSION NUMBERS

Accession numbers for Illumina reads and Affymetrix expresssion data are GSE61068 and are available here http://www.ncbi.nlm.nih.gov/geo/query/acc.cgi?acc=GSE61068.

Illumina reads are available following this link: http://www.ncbi.nlm.nih.gov/geo/query/acc.cgi?acc=GSE61066.

Affymetrix mRNA expression data are available following this link : http://www.ncbi.nlm.nih.gov/geo/query/acc.cgi?acc=GSE61067.

## SUPPLEMENTARY DATA

Supplementary Data are available at NAR Online.

SUPPLEMENTARY DATA
