# Supplementary material for: ADAR2 induces reproducible changes in sequence and abundance of mature microRNAs in the mouse brain
Source: Nucleic Acids Res. 2014 Sep 26;42(19):12155–68. doi: 10.1093/nar/gku844 (PMC4231736; doi:10.1093/nar/gku844)
Supplement: SUPPLEMENTARY DATA [file supp_42_19_12155__index.html]

ADAR2 induces reproducible changes in sequence and abundance of mature microRNAs in the mouse brain — ADAR2 induces reproducible changes in sequence and abundance of mature microRNAs in the mouse brain — SUPPLEMENTARY DATA 

# ADAR2 induces reproducible changes in sequence and abundance of mature microRNAs in the mouse brain

## SUPPLEMENTARY DATA

**Files in this Data Supplement:**

- SUPPLEMENTARY DATA
