# Supplementary material for: ADAR2 induces reproducible changes in sequence and abundance of mature microRNAs in the mouse brain
Source: Nucleic Acids Res. 2014 Sep 26;42(19):12155–68. doi: 10.1093/nar/gku844 (PMC4231736; doi:10.1093/nar/gku844)
Supplement: SUPPLEMENTARY DATA [file supp_gku844_nar-01803-a-2014-File007.pdf]

Supplementary material

A

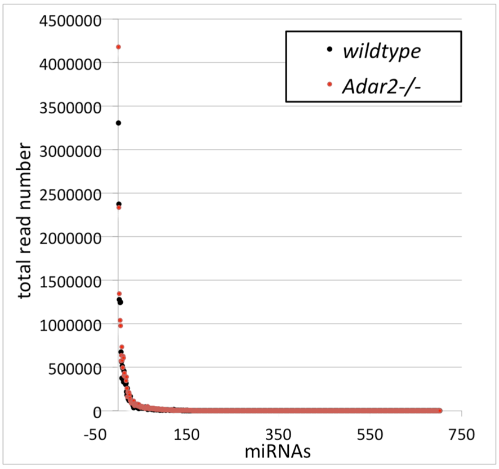

B

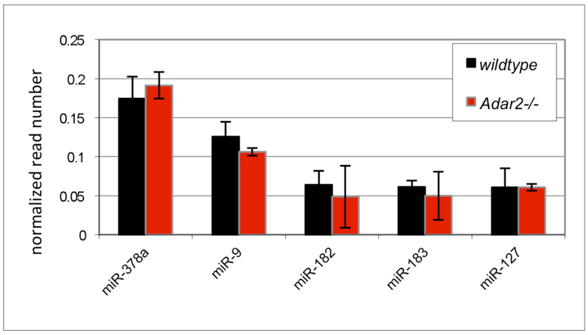

C

mmu-miRNA	avg. read no. /RPM	fold change in *Adar2$^{-/-}$*	p-value
miR-378a	3306928 / 158903	**1.11**	0.253
miR-9	2372548 / 114005	**-1.17**	0.113
miR-127	1279232667 / 61469	**1.17**	0.497
miR-182	1250965 / 60111	**-1.34**	0.321
miR-183	1242488667 / 59703	**-1.14**	0.316

Supplementary Figure S1: *Five miRNAs are highly abundant in mouse brain in all genotypes.* (a) Total read numbers of all detected miRNAs (>10 reads) in wildtype and *Adar2$^{-/-}$* mice are shown (n=3). (b) Normalized read numbers of the 5 most highly expressed miRNAs in wildtype and *Adar2$^{-/-}$* mice are plotted. One of these miRNAs, mmu-miR-378a, was found edited in this and a previous study (1). (c) None of the most highly abundant miRNAs shows a significant change in abundance between wildtype and *Adar2$^{-/-}$* mice.

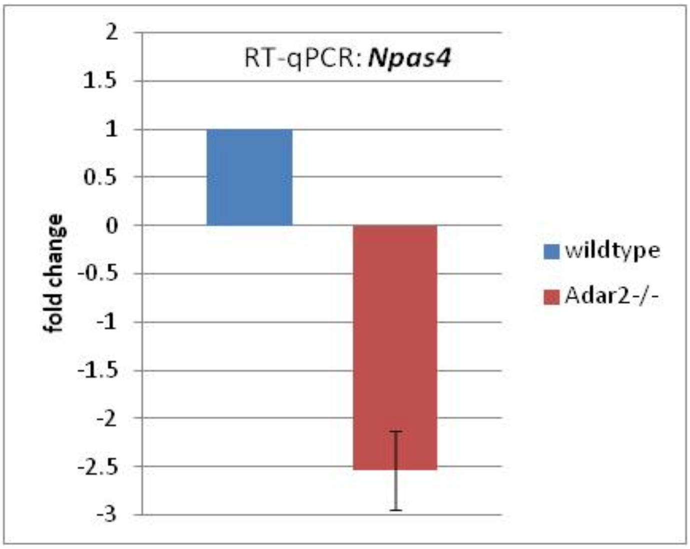

Supplementary Figure S2: *Quantitative real time PCR verifies downregulation of Npas4 mRNA in Adar2$^{-/-}$ brains.*

RT-qPCR was performed on 3 biological replicates (n=3) of total brain RNA isolated from wildtype (blue) and Adar2$^{-/-}$ (red) mice. Primers for *β-actin* were used as a reference while primers for *Npas4* were used fort he expermental RNA. Expression levels were analyzed using the $\Delta\Delta C_t$ method. Relative (*npas* to *actin*) fold-differences were calculated for wild type and Adar2$^{-/-}$ mice which shows a clear ~2.5 fold reduction of Npas4 expression in the absence of ADAR2.

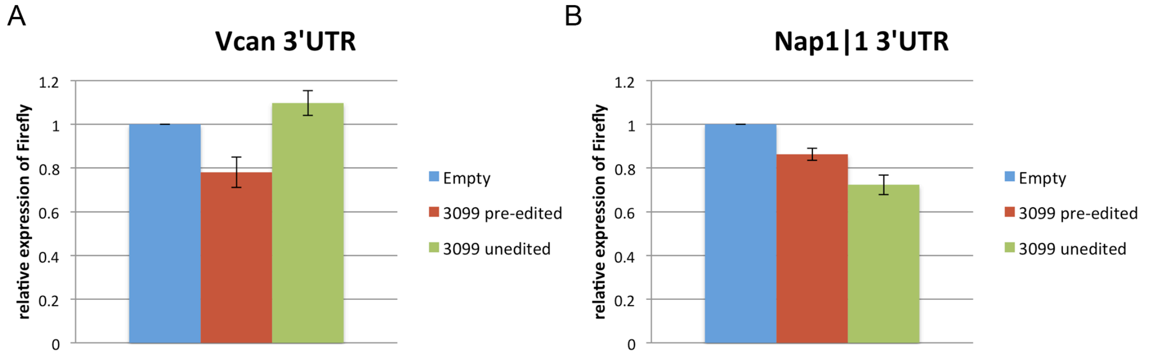

Supplementary figure S3: **Editing of miR-3099 changes its target specificity in ADAR deficient MEFs**

The 3' UTRs of *Nap1l1* – target of mmu-miR-3099, and *Vcan* – target of edited mmu-miR-3099 were introduced into pMirGlo downstream of the firefly luciferase ORF. The same plasmid also expresses Renilla luciferase as a reference (See main figure 3A for details). A) Relative Luciferase activity for the two reporter constructs was measured for the *Vcan* and B) *Nap1l1* harboring plasmids 24hrs after cotransfection with control (blue), edited (red), or unedited (green) pri-miR-3099 (n=3). The *Vcan* 3'UTR is clearly repressed by edited miR-3099 while the *Nap1l1* UTR shows a stronger response to unedited miR-3099.

KEGG pathways - Targets of unedited miR-3099
Tight junction
Adherens junction
Bacterial invasion of epithelial cells
Arrhythmogenic right ventricular cardiomyopathy (ARVC)
Dilated cardiomyopathy
**KEGG pathways - Targets of edited miR-3099**
Glutamatergic synapse
Chemokine signaling pathway
Wnt signaling pathway
Basal cell carcinoma
Axon guidance
Notch signaling pathway
D-Glutamine and D-glutamate metabolism
Nitrogen metabolism
Proximal tubule bicarbonate reclamation

Supplementary Figure S4: *KEGG* pathway analysis of predicted targets of edited and unedited miR-3099. Targets of edited miR-3099 show a clear enrichment for markers expressed during neuronal differentiation (highlighted in red). Enrichment calculation was done using the GeneCodis tool: http://genecodis.cnb.csic.es/analysis.

a

b

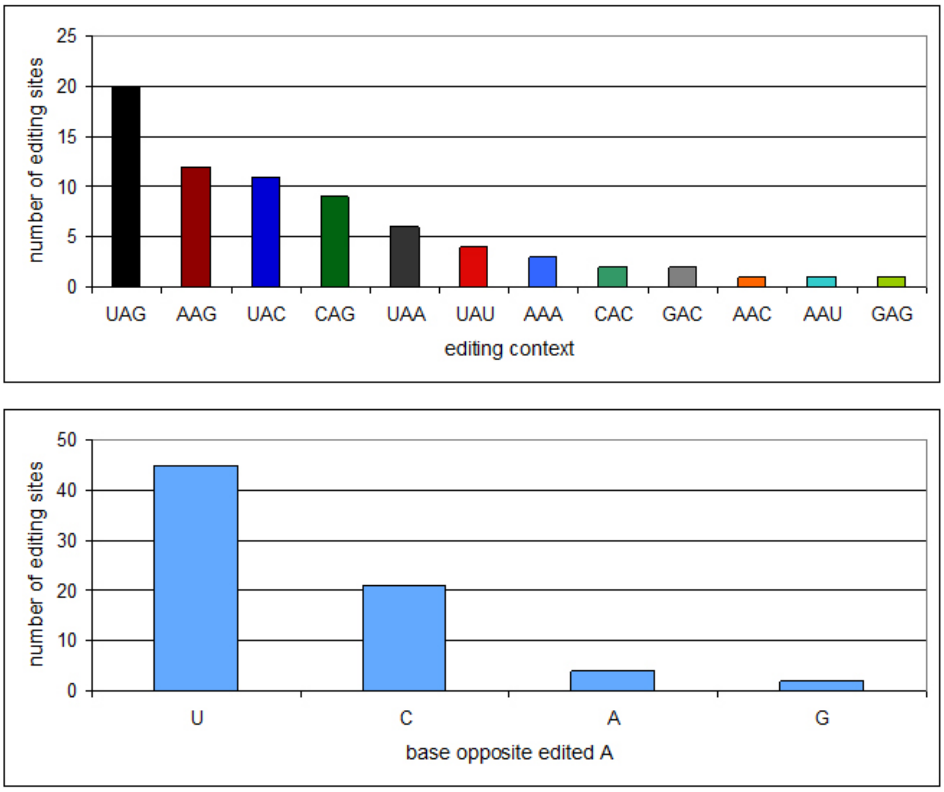

Supplementary Figure S5: *Editing context in mature miRNAs*
Editing site contexts are plotted in absolute numbers sorted by their frequency. (a) Context up-and downstream of the edited A. (b) Base opposite the edited A.

Supplementary Table S1: Consistently deregulated miRNAs with their fold changes in ADAR2 knockout brain. Underlined miRNAs were found edited in this study.

mmu-miRNA	fold change in ADAR2	p-value	avg. read number / RPM
miR-497*	-2.59	3.40E-03	2173 / 104
miR-1247*	-1.59	2.70E-03	155 / 7
miR-1298*	-1.55	4.92E-02	3463 / 166
miR-495*	-1.54	2.86E-02	267 / 13
miR-99b	-1.54	3.10E-02	167587 / 8053
miR-126-5p	-1.48	6.04E-02	85213 / 4095
miR-128-1*	-1.44	2.34E-03	1636 / 79
miR-376a*	-1.44	7.07E-02	7035 / 338
miR-129-5p	-1.43	8.05E-02	25055 / 1204
miR-1937a	-1.42	2.57E-02	223 / 11
miR-1981*	-1.41	3.94E-02	261 / 13
miR-296-3p	-1.41	2.33E-02	171 / 8
miR-30b*	-1.39	8.09E-02	766 / 37
miR-99b*	-1.39	2.01E-02	4294 / 206
miR-466i-5p	-1.37	9.54E-02	182 / 9
miR-448-3p	-1.33	7.79E-02	2344 / 113
miR-151-3p	-1.31	5.48E-02	189662 / 9113
miR-874*	-1.31	3.41E-02	956 / 46
miR-335-5p	2.76	8.41E-02	16070 / 772
miR-15b	2.46	9.41E-02	124 / 6
miR-98	2.27	9.86E-02	1911 / 92
miR-467a*	2.07	3.11E-02	266 / 13
miR-872*	2.01	8.31E-02	160 / 8
miR-467d*	1.92	3.64E-02	1033 / 50
miR-540-5p	1.90	4.75E-02	887 / 43
miR-337-3p	1.89	1.78E-02	139 / 7
miR-3107	1.88	1.78E-02	1657 / 80
miR-486	1.88	1.78E-02	1657 / 80
miR-486*	1.87	8.98E-02	431 / 21
miR-362-5p	1.86	8.54E-02	2137 / 103
miR-467e	1.83	4.35E-02	256 / 12
miR-467d	1.83	5.46E-02	3711 / 178
miR-411*	1.80	8.34E-02	944 / 45
miR-676*	1.76	3.85E-02	481 / 23
miR-376b	1.72	8.06E-02	735 / 35
let-7b	1.61	5.89E-02	116880 / 5616
miR-106b	1.60	8.27E-02	3273 / 157
miR-467a	1.56	7.96E-02	874 / 42
miR-494	1.55	9.50E-03	120 / 6
miR-155	1.55	1.07E-02	132 / 6
miR-466b-3p	1.52	9.41E-02	175 / 8
miR-466c-3p	1.52	9.41E-02	175 / 8
miR-466p-3p	1.52	9.41E-02	175 / 8
miR-489	1.51	6.45E-02	119 / 6
miR-346	1.48	8.97E-02	182 / 9
miR-672*	1.47	9.49E-02	3141 / 151

mmu-miRNA	fold change in ADAR2	p-value	avg. read number / RPM
miR-146a	1.46	9.24E-02	5902 / 284
miR-669c	1.45	9.45E-03	248 / 12
miR-129-2-3p	1.41	6.54E-02	16352 / 786
miR-592	1.40	9.54E-02	340 / 16
miR-22	1.32	8.24E-02	32003 / 1538
miR-467c	1.32	9.89E-02	209 / 10
miR-879	1.32	7.68E-02	2295 / 110
miR-679-5p	1.31	1.53E-02	595 / 29
miR-873	1.30	8.76E-02	1155 / 55

Supplementary Table S2: *Significant T to C transition events in mature miRNAs.*
Test for significance in *Adar2*$^{-/-}$ but not in wildtype. The column „frequency (%)“ shows the frequency of a C instead of a T at the respective position. The position in the mentioned miRNA, the number of total reads for this miRNA, the standard deviation (SD) for three replicates, and the sequence specific context before and after the altered U and the position opposite the altered U are given in “context”.

mmu-miRNA	position	read number	frequency (%)	SD	context
miR-126-5p	16	72386	**0.18**	0.044	GUA/A
miR-429	14	37253	**0.22**	0.038	GUA/A
miR-1	8	30148	**0.20**	0.031	GUA/A
let-7f	6	18521	**0.35**	0.103	GUA/A
miR-194	19	15315	**0.25**	0.043	GUG/A
miR-341	17	11517	**0.21**	0.032	GUC/G
miR-181c*	17	7428	**0.30**	0.004	GUG/A
miR-1839-5p	18	4601	**0.32**	0.074	GUC/A
miR-139-5p	8	2584	**0.43**	0.051	GUG/C
miR-374	21	1860	**0.54**	0.050	GUG/A
miR-20a	21	1840	**0.66**	0.130	GUA/A
miR-300*	18	1762	**0.29**	0.069	CUU/G
miR-3057-5p	15	1175	**0.69**	0.191	UUC/G
miR-212-3p	14	1117	**0.61**	0.111	GUC/G
miR-3470b	19	376	**1.87**	0.509	CUG/U

Supplementary Table S3: All detected editing events in mature mouse brain miRNAs sorted by % editing in wildtype. Preferential editing by either ADAR2 or ADAR1 are rated by number of „x“ in the respective columns; with „x“ meaning: preferential editing by the enzyme, and „xxx“ meaning: exclusively edited by the enzyme.

mmu-miRNA	position	context	Wt % edit	SD	Adarb1$^{-/-}$ edit	SD	Fold change editing	preftl. editing by Adarb1	preftl. editing by ADAR1	avg. read number / RPM	Reference
**miR-3099**	7	UAG/C	**79.8**	6.31	**66**	5.03	-1.2			2359 / 113	[1-3]
**miR-497***	20	UAC/C	**73.2**	2.49	**3**	0.88	-24.1	xxx		1560 / 75	[3]
**miR-411**	5	UAG/C	**68.4**	5.61	**42.2**	4.11	-1.6	x		1451 / 70	[2-4]
**miR-376a***	4	UAG/C	**58.5**	4.75	**6.7**	0.9	-8.7	xx		6104 / 293	[2-4]
**miR-497**	2	CAG/U	**52.3**	2.05	**1**	0.32	-54.1	xxx		1266 / 61	[3]
**miR-467c**	3	AAG/A	**48.2**	3.36	**15**	2.71	-3.2	x		259 / 12	this study
**miR-542-3p**	16	AAC/G	**47.7**	4.98	**63.1**	2.94	1.3		x	464 / 22	this study
**miR-379**	5	UAG/C	**46.4**	4.55	**11.7**	0.44	-4	x		103648 / 4980	[2-4]
**miR-376b**	6	UAG/U	**45.4**	2.86	**66.1**	4.42	1.5		xx	1089 / 52	[1, 3, 4]
**miR-376c**	6	UAG/U	**40.3**	5.67	**42.1**	1.22	1			1092 / 52	[2-4]
**let-7e**	19	UAG/U	**29.1**	11.93	**42.7**	10.46	1.5		xx	5152 / 248	this study
**miR-467e**	4	AAG/C	**26.3**	3.12	**10.6**	0.5	-2.5	x		393 / 19	this study
**miR-99b***	3	AAG/U	**15.4**	1.56	**10.8**	1.3	-1.4			3891 / 187	[4]
**miR-467d**	3	AAG/C	**15.2**	1.11	**10.5**	0.84	-1.4			5331 / 256	[3]
**miR-381**	4	UAC/U	**13.7**	2.61	**23**	2.53	1.7		xx	64443 / 3097	[1-3]
**miR-574-5p**	17	GAG/U	**13.2**	3.89	**15.3**	6.84	1.2			122 / 6	this study
**miR-540-5p**	3	AAG/U	**12.1**	1.19	**0.2**	0.15	-52.1	xxx		1405 / 68	[3]
**miR-421**	14	UAA/A	**10.9**	1.96	**3.3**	0.33	-3.3	x		1384 / 67	[3]
**miR-3099**	2	UAG/C	**9.6**	1.21	**8.2**	1.16	-1.2			2359 / 113	this study
**miR-467a**	3	UAA/U	**9.2**	1.45	**4.8**	0.14	-1.9	x		1157 / 56	[2]
**miR-378a**	16	CAG/U	**6.3**	0.68	**0.2**	0.01	-34.4	xxx		3743784 / 179893	[1, 2]
**miR-708***	21	UAG/A	**5.6**	1.3	**0.4**	0.04	-12.9	xx		14031 / 674	this study

mmu-miRNA	position	context	Wt % edit	SD	Adarb1$^{-/-}$ edit	SD	Fold change editing	preftl. editing by Adarb1	preftl. editing by ADAR1	avg. read number / RPM	Reference
**miR-411**	2	UAG/U	**5**	1.26	**1.1**	0.15	-4.7	xx		1451 / 70	this study
**let-7e**	17	UAU/U	**4.9**	2.42	**7.1**	1.34	1.5		xx	5152 / 248	[2]
**miR-467d***	9	UAC/C	**4.8**	0.44	**10.1**	2.91	2.1		xx	1598 / 77	[1, 2]
**miR-301b**	20	AAA/C	**4.8**	1.28	**1.6**	1.33	-3	x		201 / 10	this study
**miR-3061-3p**	3	UAC/U	**4.2**	1.29	**7.5**	0.98	1.8		xx	165 / 8	this study
**miR-669c**	3	UAG/U	**3.9**	1.15	**0.9**	0.16	-4.5	xx		323 / 16	this study
**miR-488**	5	AAA/C	**3.1**	0.13	**2.9**	0.44	-1.1			5993 / 288	this study
**miR-151-3p**	3	UAG/U	**2.8**	1.62	**4.4**	0.71	1.6		x	172895 / 8308	[4]
**miR-3086-5p**	2	UAG/U	**2.7**	0.3	**0.2**	0.02	-17.6	xx		4736 / 228	this study
**miR-376b***	7	UAU/C	**2.7**	0.05	**0.4**	0.23	-6.4	xx		34919 / 1678	this study
**miR-154**	2	UAG/U	**2.5**	0.64	**0.1**	0.02	-25.8	xxx		7473 / 359	this study
**miR-384-5p**	4	UAA/C	**2.4**	0.15	**1**	0.06	-2.5	x		2167 / 104	this study
**miR-24**	21	CAG/G	**2.4**	0.49	**0.1**	0	-38.8	xxx		177863 / 8546	this study
**miR-540-5p**	9	CAC/C	**2.4**	1.25	**0.2**	0.09	-11.5	xx		1405 / 68	this study
**miR-301a**	20	AAA/C	**2.4**	0.48	**0.1**	0.07	-19.6	xx		5391 / 259	[2]
**miR-770-5p**	4	CAC/A	**2.4**	0.88	**0.8**	0.21	-2.9			2081 / 100	this study
**miR-466a-3p**	4	UAC/U	**2.3**	0.55	**2.3**	0.42	1			438 / 21	this study
**miR-466e-3p**	4	UAC/U	**2.3**	0.55	**2.3**	0.42	1			438 / 21	this study
**miR-467e**	3	UAA/U	**2.1**	0.65	**2.1**	0.44	1			393 / 19	this study
**let-7d***	5	UAC/C	**1.9**	1.07	**0**	0	0			218 / 10	this study
**miR-421**	7	CAG/U	**1.9**	0.13	**1.5**	0.27	-1.3			1384 / 67	this study
**miR-381**	7	AAG/U	**1.9**	0.12	**2.3**	0.06	1.2		x	64443 / 3097	this study
**miR-708***	6	UAG/U	**1.8**	0.24	**1.7**	0.25	-1.1			14031 / 674	this study
**miR-195**	5	CAG/U	**1.7**	0.3	**0**	0.02	-42.9	xxx		21647 / 1040	this study
**miR-219-3p**	15	GAC/U	**1.7**	1.56	**2.5**	1.71	1.5		xx	17700 / 851	[3]
**miR-24-2***	7	UAC/C	**1.6**	0.56	**0**	0.03	-41.2	xxx		1262 / 61	[2]
**miR-335-5p**	4	AAG/C	**1.5**	0.34	**0.8**	0.08	-1.7	x		32768 / 1575	this study

mmu-miRNA	position	context	Wt % edit	SD	Adarb1$^{-/-}$ edit	SD	Fold change editing	preftl. editing by Adarb1	preftl. editing by ADAR1	avg. read number / RPM	Reference
**miR-137**	16	UAC/U	**1.4**	0.38	**0.1**	0.02	-11.5	xx		15990 / 768	this study
**miR-222**	15	UAC/U	**1.3**	0.23	**0.4**	0.12	-3.2	x		3976 / 191	this study
**miR-184**	17	UAA/U	**1.3**	0.57	**1.4**	0.43	1.1			1112 / 53	this study
**miR-873**	3	CAG/C	**1.3**	0.69	**0.2**	0.06	-7.4	xx		1417 / 68	this study
**miR-98**	11	AAG/U	**1.2**	0.89	**1.2**	0.29	1			3236 / 155	[2]
**let-7d**	19	UAG/U	**1.1**	0.43	**0.8**	0.27	-1.4			2969 / 143	this study
**miR-467d**	2	UAA/U	**1.1**	0.15	**0.7**	0.08	-1.5	x		5331 / 256	this study
**miR-300**	7	AAG/U	**1**	0.08	**0.3**	0.02	-3.4	x		11284 / 542	this study
**miR-488**	6	AAG/U	**0.9**	0.18	**0.9**	0.18	1			5993 / 288	this study
**miR-338-3p**	4	CAG/U	**0.9**	0.15	**0.1**	0.01	-13.6	xx		7953 / 382	this study
**miR-467d***	5	UAC/U	**0.8**	0.21	**0.8**	0.23	1			1598 / 77	this study
**miR-203**	21	UAG/C	**0.7**	0.13	**0**	0.02	-23	xx		1941 / 93	[4]
**miR-101b**	4	CAG/U	**0.7**	0.32	**0.2**	0.04	-3.9	x		222048 / 10670	this study
**miR-708**	2	AAG/U	**0.7**	0.08	**0.6**	0.05	-1.1			33924 / 1630	this study
**miR-98**	17	UAU/U	**0.6**	0.07	**0.8**	0.38	1.3		x	3236 / 155	this study
**miR-130a**	8	AAU/U	**0.6**	0.09	**0.9**	0.36	1.6		xx	8972 / 431	[2]
**miR-378a**	6	GAC/U	**0.5**	0.25	**0.4**	0.12	-1.4			3743784 / 179893	this study
**miR-130a**	16	AAG/U	**0.5**	0.1	**0.1**	0.02	-5.6	xx		8972 / 431	this study
**miR-140**	16	UAU/C	**0.5**	0.08	**0.1**	0.02	-7.6	xx		17240 / 828	this study
**let-7f**	19	UAG/U	**0.4**	0.14	**0.3**	0.05	-1.5	x		18520 / 890	this study
**miR-29b**	2	UAG/U	**0.4**	0.07	**0.2**	0.03	-1.6	x		42057 / 2021	this study
**miR-29c**	2	UAG/U	**0.3**	0.02	**0.1**	0.01	-4	x		51535 / 2476	this study
**let-7g**	19	CAG/U	**0.3**	0.05	**0.4**	0.09	1.2			88633 / 4259	this study

References for Supplementary material

1. Vesely, C., Tauber, S., Sedlazeck, F.J., von Haeseler, A. and Jantsch, M.F. (2012) Adenosine deaminases that act on RNA induce reproducible changes in abundance and sequence of embryonic miRNAs. Genome Research, 22, 1468-1476.
2. Ekdahl, Y., Farahani, H.S., Behm, M., Lagergren, J. and Öhman, M. (2012) A-to-I editing of microRNAs in the mammalian brain increases during development. Genome Research, 22, 1477-1487.
3. Chiang, H.R., Schoenfeld, L.W., Ruby, J.G., Auyeung, V.C., Spies, N., Baek, D., Johnston, W.K., Russ, C., Luo, S., Babiarz, J.E. et al. (2010) Mammalian microRNAs: experimental evaluation of novel and previously annotated genes. Genes & Development, 24, 992-1009.
4. Kawahara, Y., Megraw, M., Kreider, E., Iizasa, H., Valente, L., Hatzigeorgiou, A.G. and Nishikura, K. (2008) Frequency and fate of microRNA editing in human brain. Nucleic Acids Research, 36, 5270-5280.
